# Supplementary figures and images for: Engineered red blood cells carrying PCSK9 inhibitors persistently lower LDL and prevent obesity
Source: PLoS One. 2021 Nov 3;16(11):e0259353. doi: 10.1371/journal.pone.0259353 (PMC8565730; doi:10.1371/journal.pone.0259353)

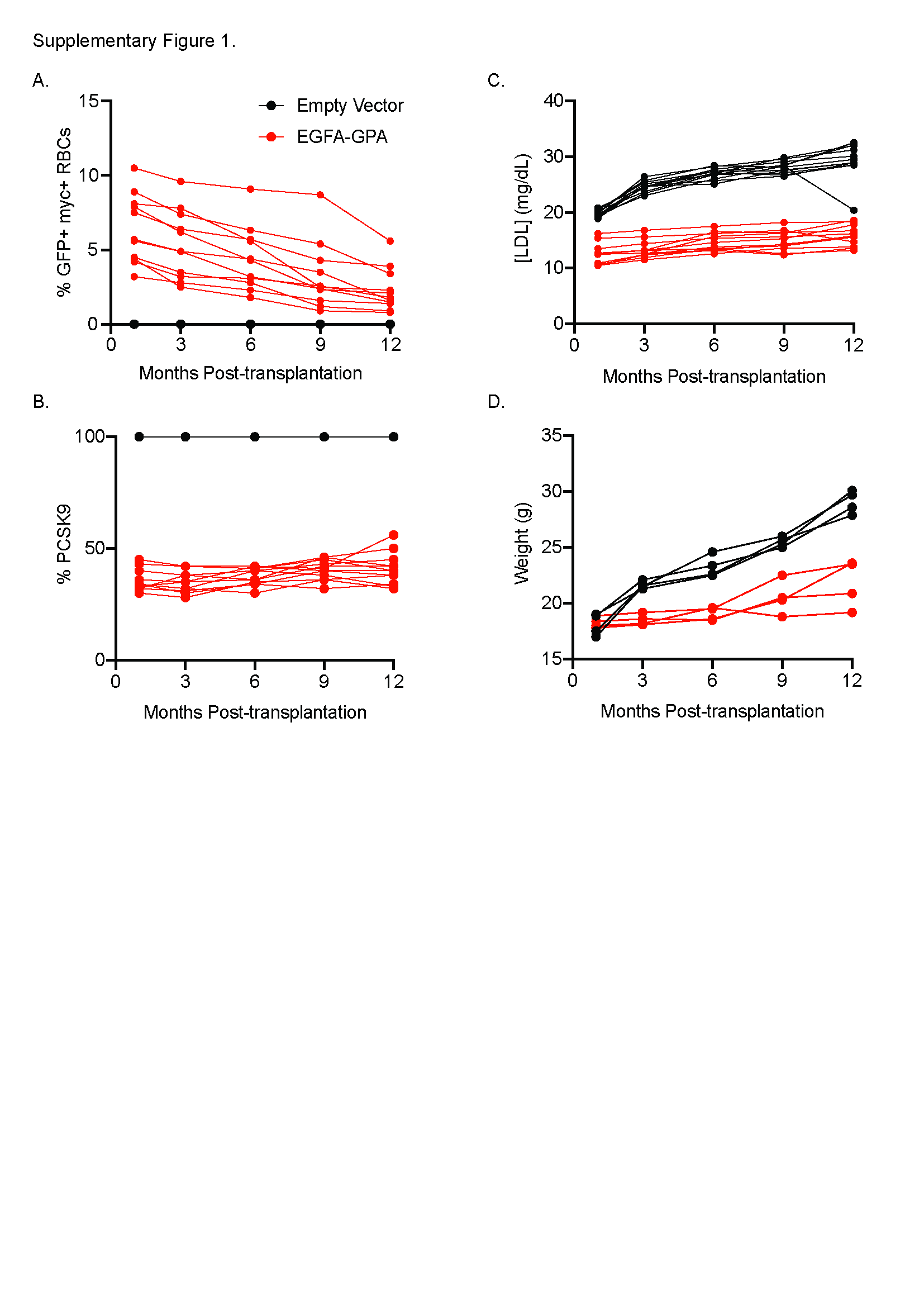

Supplement: S1 Fig — (TIFF) [file pone.0259353.s001.tiff]

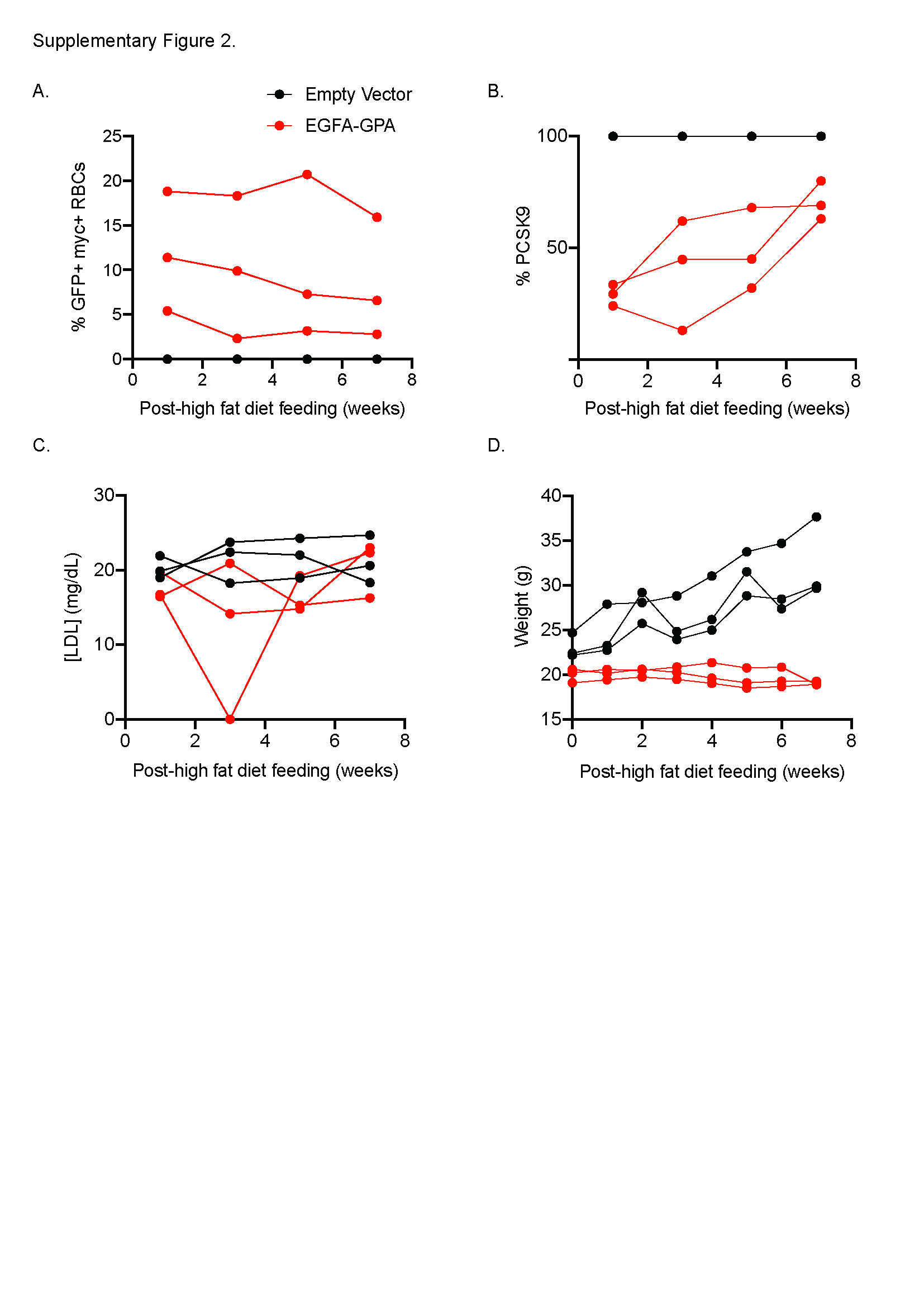

Supplement: S2 Fig — (TIFF) [file pone.0259353.s002.tiff]

Supplementary Figure 3.

A.

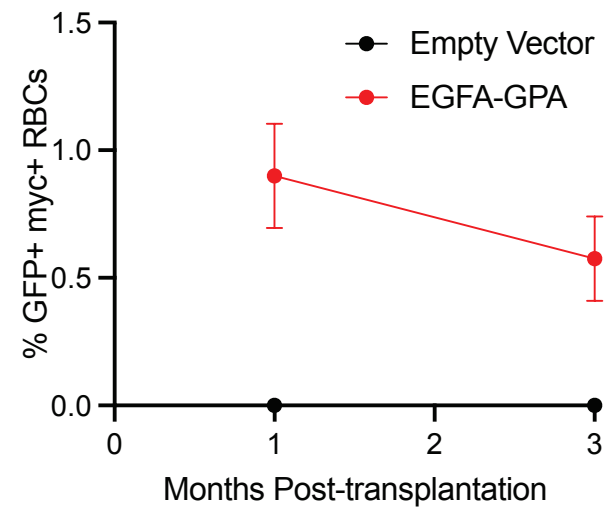

B.

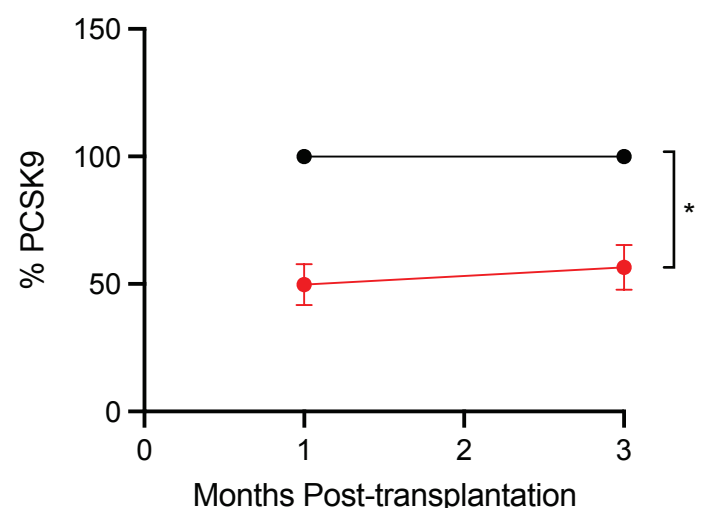

C.

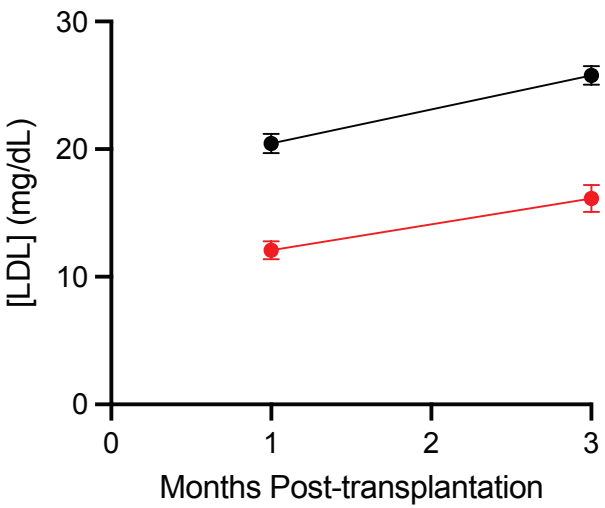

D.

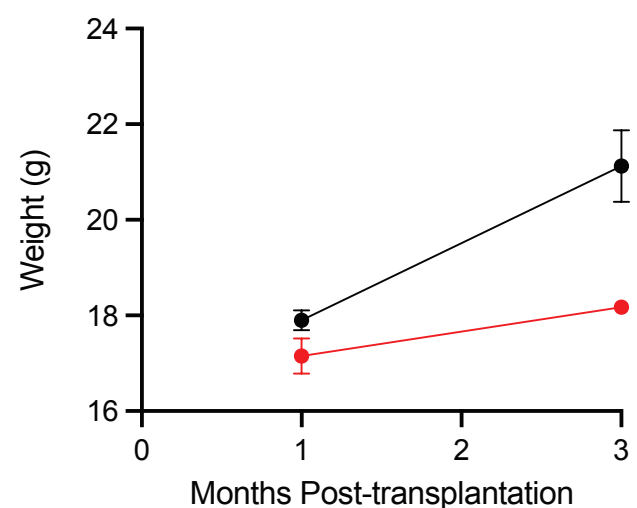

E.

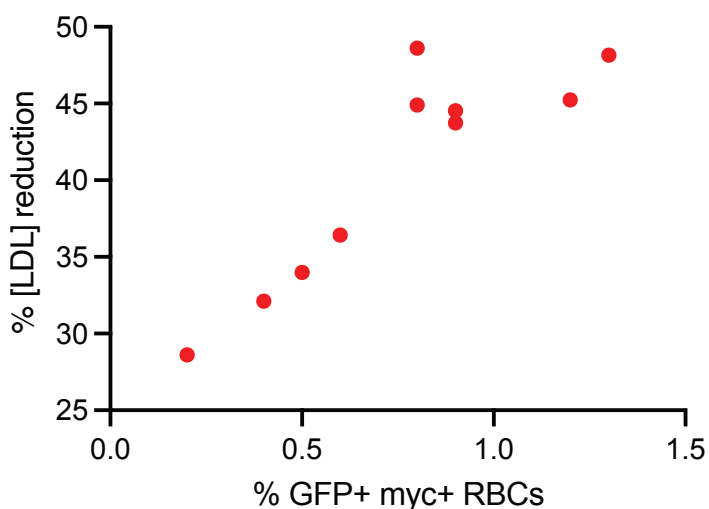

Supplement: S3 Fig — (A) Expression of myc+ cells (n = 4, mean ± SEM) in mice transplanted with a reduced number of HSPCs expressing the Glycophorin A- EGF-A chimeric protein. (B) Plasma PCSK9 and (C) LDL levels of transplanted mice were quantified via ELISA assay at the indicated time points. (D) weight of mice expressing EGFA-GPA RBCs. (n = 4, mean ± SEM) (E) Correlation of % GFP-EGFA expression with LDL reduction. (PDF) [file pone.0259353.s003.pdf]
